# Correction: A hierarchical Ca/TiO_2_/NH_2_-MIL-125 nanocomposite photocatalyst for solar visible light induced photodegradation of organic dye pollutants in water

**DOI:** 10.1039/d3ra90057f

**Published:** 2023-06-29

**Authors:** Najmeh Ahmadpour, Mohammad Hossein Sayadi, Shahin Homaeigohar

**Affiliations:** a Department of Environmental Engineering, Faculty of Natural Resources and Environment, University of Birjand Birjand Iran mh_sayadi@birjand.ac.ir +98 5632254066 +98 5632254068; b School of Science & Engineering, University of Dundee Dundee DD1 4HN UK; c Nanochemistry and Nanoengineering, Department of Chemistry and Materials Science, School of Chemical Engineering, Aalto University Kemistintie 1 00076 Aalto Finland

## Abstract

Correction for ‘A hierarchical Ca/TiO_2_/NH_2_-MIL-125 nanocomposite photocatalyst for solar visible light induced photodegradation of organic dye pollutants in water’ by Najmeh Ahmadpour *et al.*, *RSC Adv.*, 2020, **10**, 29808–29820, https://doi.org/10.1039/D0RA05192F.

The authors regret that an incorrect grant number was shown in the Acknowledgements section of the published article. The corrected section should read:

“N. A. and M. H. S. gratefully acknowledge the Research Council of University of Birjand (Grant Number: 9945/1399) for the financial support…”

The Royal Society of Chemistry apologises for these errors and any consequent inconvenience to authors and readers.

## Supplementary Material

